# Eosinophilic Gastroenteritis in the Small Intestine Mimicking Eosinophilic Granulomatosis With Polyangiitis in a Young Male Patient

**DOI:** 10.7759/cureus.29813

**Published:** 2022-10-01

**Authors:** Maika Hayashi, Ryuichi Ohta, Fumiko Yamane, Tsuyoshi Mishiro, Chiaki Sano

**Affiliations:** 1 Family Medicine, Shimane University Faculty of Medicine, Izumo, JPN; 2 Community Care, Unnan City Hospital, Unnan, JPN; 3 Internal Medicine, Unnan City Hospital, Unnan, JPN; 4 Community Medicine Management, Shimane University Faculty of Medicine, Izumo, JPN

**Keywords:** japan, family medicine, rural hospital, eosinophilic granulomatosis with polyangiitis, small intestine, eosinophilic gastroenteritis

## Abstract

Eosinophilic gastroenteritis (EGE) is a disease involving the gastrointestinal tract that involves the invasion of eosinophils into the lumen of the tract. Invasion of eosinophils in the tract causes edema and inflammation of the wall, resulting in acute or chronic diarrhea and abdominal pain. EGE can cause chronic inflammation in the small intestine, thereby mimicking chronic vasculitis. We report the case of a 24-year-old male patient with the chief complaint of acute progressive abdominal pain. Endoscopic findings of the small intestine were chronic and similar to those of polyangiitis (eosinophilic granulomatosis with polyangiitis). This case shows the presence of various clinical courses of EGE and the discrepancy between the symptoms and endoscopic appearance of vasculitis. Physicians should focus not only on acute symptoms but also on chronic phases of the disease to prevent disease progression and modify patients’ help-seeking behaviors according to their symptoms.

## Introduction

Eosinophilic gastroenteritis (EGE) is a disease involving the gastrointestinal tract and the invasion of eosinophils into the lumen of the gastrointestinal tract [1]. Invasion of eosinophils into the tract causes edema and inflammation of the wall, which causes acute or chronic diarrhea and abdominal pain [1,2]. The symptoms and locations can differ according to the location of the lesions in the gastrointestinal tract [2]. The main lesions involve the stomach and duodenum and can cause epigastric pain, nausea, and vomiting [1]. When the inflammation caused by eosinophils spreads to the small intestine, the main symptoms are diarrhea and umbilical and lower abdominal pain. This disease is rare and prevalent among younger generations [3].

EGE may cause acute and chronic inflammation localized in the small intestine, similar to vasculitis in endoscopic findings. This type of disease is rare and has not been frequently described in the clinical course [4,5]. We encountered a young male patient with EGE who progressed rapidly during the clinical course. However, endoscopic findings of the small intestine are chronic and similar to eosinophilic granulomatosis with polyangiitis (EGPA). This case shows the presence of various clinical courses of EGE and the discrepancy between the symptoms and endoscopic appearance of vasculitis.

## Case presentation

A 24-year-old male patient presented to our community hospital's outpatient department with chief complaints of several days of fever, epigastric pain, and diarrhea. The patient had consumed barbeque 10 days earlier and raw fish eight days earlier. He was suspected of having gastroenteritis and was treated with intravenous hydration and discharged. Seven days later, the patient visited our hospital again because of a flare-up of the same symptoms. He had consumed beef stew for dinner four hours prior to the visit. The patient reported that his abdominal pain was worse and more intense than on his previous visit. He had a past medical history of atopic dermatitis (AD). His medication included a second-generation antihistamine before the first and second visits.

**Figure 1:**
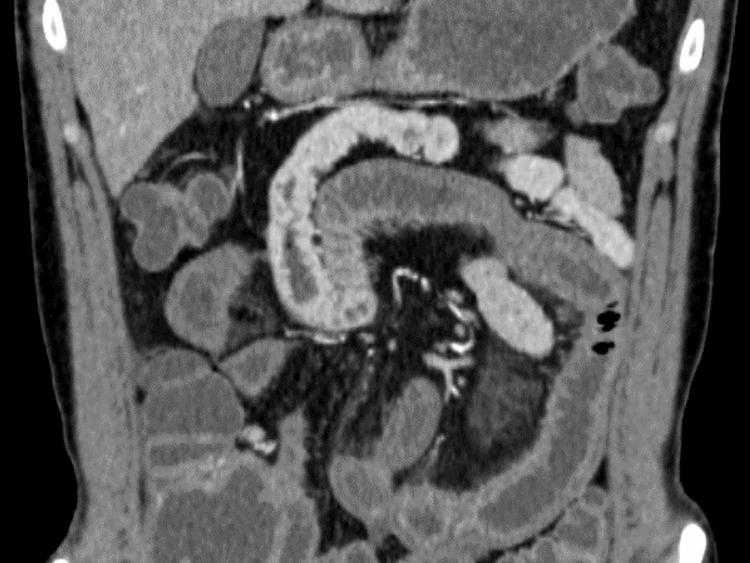
Abdominal computed tomography with contrast shows extensive edematous wall thickening of the small intestine

At the time of admission, his vital signs were as follows: blood pressure 121/71 mmHg; pulse rate 71 beats/min; body temperature 37.2 °C; respiratory rate 18 breaths/min; and oxygen saturation 97% on room air. He was aware of time, place, and person. Physical examination revealed gross abdominal tenderness with tapping pain. There were no obvious abnormalities in the chest; however, urticaria was observed in the chest and abdominal skin. Abdominal ultrasound showed ascites and dilatation of the small intestine. Contrast-enhanced abdominal computed tomography (CT) revealed extensive edematous wall thickening of the small intestine (Figure 1).

Because hereditary angioedema, vasculitis, and lupus enteritis have been suggested as differential diagnoses, C1 inhibitors, antineutrophil cytoplasmic antibodies, and antinuclear antibodies were added (Table 1).

**Table 1:** Initial laboratory data during the hospitalization

Marker	Level	Reference
White blood cells	12.60	3.5–9.8 × 10^3^/μL
Neutrophils	68.8	44.0%–72.0%
Lymphocytes	20.3	18.0%–59.0%
Monocytes	6.0	0.0%–12.0%
Eosinophils	4.6	0.0%–10.0%
Basophils	0.3	0.0%–3.0%
Red blood cells	4.93	3.76–5.50 × 10^6^/μL
Hemoglobin	15.3	11.3–15.2 g/dL
Hematocrit	45.0	33.4%–44.9%
Mean corpuscular volume	91.3	79.0–100.0 fl
Platelets	20.3	13.0–36.9 × 10^4^/μL
Total protein	7.3	6.5–8.3 g/dL
Albumin	4.8	3.8–5.3 g/dL
Total bilirubin	1.3	0.2–1.2 mg/dL
Aspartate aminotransferase	21	8–38 IU/L
Alanine aminotransferase	20	4–43 IU/L
Alkaline phosphatase	58	106–322 U/L
γ-glutamyl transpeptidase	20	<48 IU/L
Lactate dehydrogenase	213	121–245 U/L
Blood urea nitrogen	12.9	8–20 mg/dL
Creatinine	1.07	0.40–1.10 mg/dL
Estimated glomerular filtration rate	72.3	> 60.0 mL/min/L
Serum Na	141	135–150 mEq/L
Serum K	3.5	3.5–5.3 mEq/L
Serum Cl	105	98–110 mEq/L
Immunoglobulin E	937	< 173 IU/mL
Anti-nuclear antibodies	<40	< 40
Proteinase 3-ANCA	<1.0	< 3.5 U/mL
Myeloperoxidase-ANCA	<1.0	< 3.5 U/mL
C1 inactivator activity	122	70%–130%

**Figure 2:**
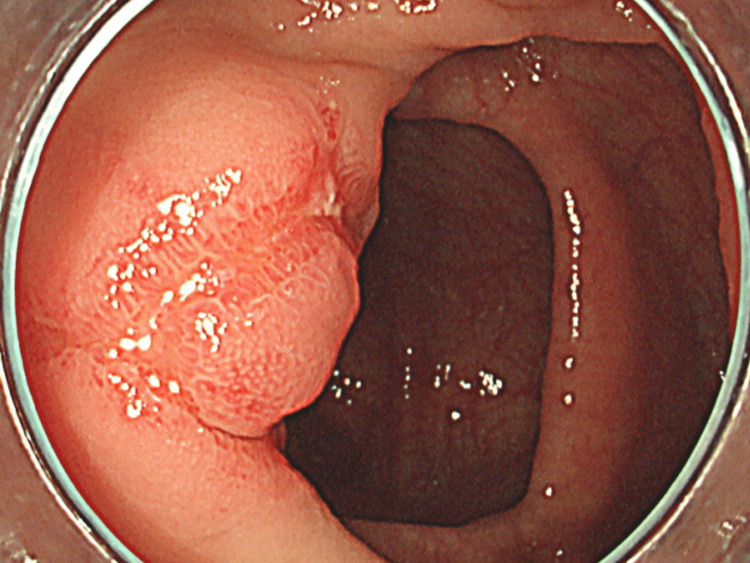
Colonoscopy shows a linear ulcer with a ring-shaped structure at the terminal ileum

Because the edematous wall thickening also extended to the ileum, a colonoscopy was performed for further investigation. Colonoscopy revealed a ring-shaped linear ulcer in the terminal ileum. Eosinophilic granulomatosis with polyangiitis (EGPA) was suspected, and a biopsy was performed (Figure 2).

Because of wall thickening of the small intestine on CT, small bowel endoscopy was performed, which revealed gross edema and erythema in the lumen.

**Figure 3:**
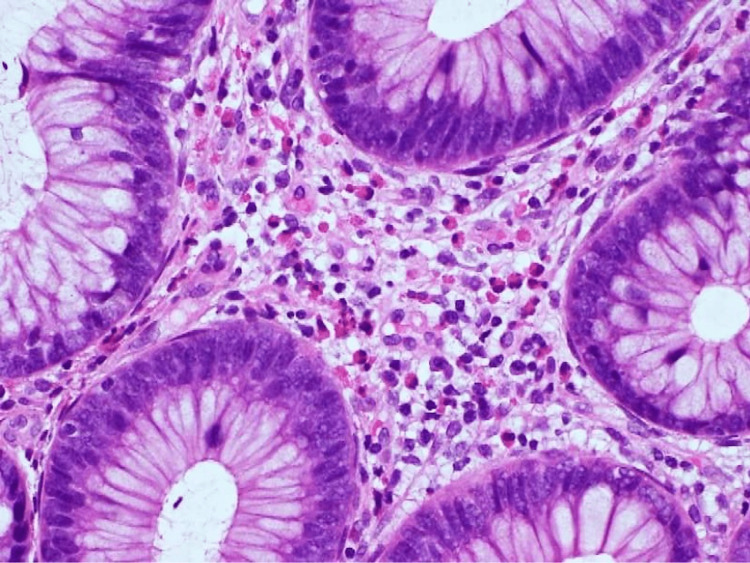
Pathological finding of the biopsy of the terminal ileum showing the invasion of a plethora of eosinophils to the wall

Additional blood tests were negative for hereditary angioedema, lupus enteritis, and vasculitis (Table 1). Eosinophils were detected at >50/HPF in the biopsy of the terminal ileum, and a few eosinophils were detected in the sigmoid colon and rectum (Figure 3).

The biopsy results of the small intestine endoscopy showed 10-15 eosinophils/HPF. On admission, because the patient had eosinophilia (579/μL (normal range, 100 to 300)), elevated immunoglobulin E levels, and numerous eosinophils in the biopsy of the intestinal lumen, he was diagnosed with eosinophilic enterocolitis. The patient was treated with rehydration, and his abdominal pain became less severe. Six days after admission, he was discharged with a prescription for pranlukast hydrate (leukotriene receptor antagonist).

## Discussion

This case report describes how EGE shows endoscopic findings of EGPA and how the symptoms can appear suddenly with a self-limited clinical course. General physicians should carefully consider the possibility of EGE in the clinical course and the requirement for continuous treatment in young patients.

EGE can reveal endoscopic findings of EGPA. The pathophysiology of EGE and EGPA is similar [5,6]. Both diseases are triggered by allergic reactions in their pathophysiology [7]. EGPA is a systemic vasculitis that causes continual inflammation in systemic organs; therefore, an inflammatory reaction can be detected using clinical symptoms and laboratory data [8]. On the other hand, EGE is based on the allergic reaction triggered by eosinophilia and elevated immunoglobulin E levels [8,9]. Transient inflammation can appear but disappear during the clinical course. The initial clinical courses of these diseases may differ.

The chronic phases of these diseases are similar, particularly in the gastrointestinal organs. Without treatment, EGE causes relapsing inflammation of the gastrointestinal tract wall caused by a type 1 allergic reaction [1]. Chronic relapsing inflammation may cause changes in mucus in the gastrointestinal tract. Endoscopic findings can differ in the initial phases of EGE and EGPA. However, the involvement of chronic relapsing inflammation may change the endoscopic findings of EGE, resembling EGPA and vasculitis, as seen in our case [3,4]. Endoscopically diagnosing EGE and ruling out EGPA can be challenging. Therefore, physicians should take precise clinical histories and conduct proper physical examinations to differentiate between these diseases. Moreover, the progression of EGE could cause vasculitis such as EGPA; therefore, physicians should follow their symptoms and further investigate the exacerbation to search for vasculitis [4].

The clinical course of EGE can include both acute and chronic findings, regardless of symptom severity. Existing literature shows that the symptoms of EGE can appear suddenly in the gastrointestinal tract in a self-limiting manner [10]. The apparent symptoms were transient and resolved. However, our case shows that EGE may progress without symptoms based on endoscopic and pathological findings. Chronic changes in the lumen of the gastrointestinal tract can cause various complications such as chronic constipation, diarrhea, and abdominal pain [3]. Inflammation in the GI tract due to EGE or EGPA can lead to cancer, as other inflammatory gastrointestinal diseases such as Crohn’s disease and ulcerative colitis [11,12]. Physicians may focus on the present symptoms and treatments while patients may insist on the present symptoms without a previous chronic clinical course. In diagnosing EGE, physicians and patients should also focus on chronic clinical courses to prevent the progression of the disease to cancer and vasculitis due to continual inflammation and modify their help-seeking behaviors according to their symptoms [13,14]. Patients with EGE can be facilitated to control their symptoms by controlling their food. and notice and discuss their systemic symptoms with their primary care physicians. The symptoms may be progressive, and they should use their primary care physicians efficiently to prevent additional inflammation of the guts.

## Conclusions

We experienced a transient and rapid course of EGE mimicking EGPA. EGE can be based on types 1 and 3 allergies with a rapid onset course, and inflammation may be localized in the small intestine. An appropriate pathological evaluation based on the patient’s clinical history is important. A precise understanding of the clinical symptoms and course of EGE is essential to prevent disease progression and modify patients’ help-seeking behaviors according to their symptoms.
